# Hypohyperodontia in a sample of Chinese population: a retrospective study

**DOI:** 10.1186/s12903-025-07213-6

**Published:** 2025-12-01

**Authors:** Shuang Li, Xiaojie Zhou, Guoxia Yu

**Affiliations:** Department of Stomatology, National Center for Children’s Health, Beijing Children’s Hospital, Capital Medical University, Beijing, China

**Keywords:** Concomitant hypodontia and hyperdontia, Hypo-hyperdontia, Hypohyperdontia, Oligopleiodontia, Panoramic radiograph

## Abstract

**Background:**

Hypohyperdontia, a rare numeric dental anomaly combining tooth agenesis and supernumerary teeth (ST), impacts aesthetics and function. Limited epidemiological data exist in Chinese populations. This study investigates its prevalence and distribution.

**Methods:**

This retrospective study analyzed 10,112 panoramic radiographs(PRs)(August 2021 ~ December 2023) to assess hypohyperdontia prevalence, distribution and associated characteristics, including patient demographics, dentition type, ST (number, morphology, location, orientation), and tooth agenesis patterns (number, location). The hypohyperdontia patients were stratified into pre- and post-eruption groups based on permanent anterior/supernumerary tooth eruption status. All data were presented as counts(n) and percentage (%), and statistically analysed using logistic regression and Chi-square test.

**Results:**

Prevalence was 0.66% (67/10112; M: F 42:25) without gender difference (OR = 1.28, 95% CI: 0.78–2.11, *p* = 0.329). Most cases were diagnosed in the mixed dentition (mean age: 6.97 years), with 85.07% (57/67) affecting both jaws. The anteroposterior type (58.21%, 39/67) predominated over anterior type (41.79%, 28/67). Eruption-wise, 10 and 57 cases were pre- and post-eruption, respectively. ST were predominantly single (73.13%, 49/67), conical (56/85), and vertically/inverted-oriented (38/85 and 34/85, respectively), with premaxillary predilection (*p* = 0.001). Single-tooth agenesis was most common (38/67 cases; total missing teeth = 115). Mandibular agenesis was more frequent (*p* = 0.001), primarily affecting second premolars (64/115), followed by lateral incisors (26/115), without side preference (*p* = 0.641).

**Conclusions:**

Hypohyperdontia prevalence is 0.66%, within the global range (0.002% to 3.1%) with no gender bias. Typically diagnosed in mixed dentition, most cases are bimaxillary involvement. Isolated posterior involvement is rare. ST and agenesis patterns mirror isolated anomalies. Early panoramic screening in suspected cases facilitates timely multidisciplinary management to prevent functional and aesthetic complications.

## Background

Numeric dental anomalies include tooth agenesis (hypodontia) and supernumerary teeth (ST) (hyperdontia). The simultaneous occurrence of both conditions in a single individual—termed hypohyperdontia—is exceptionally rare. Initially described by Camilleri [1] as “concomitant hypodontia and hyperdontia,” the condition was later termed hypohyperdontia by Gibson, which has since become the standard nomenclature [2]. Genetic factors are predominant in hypohyperdontia pathogenesis, though environmental influences can modify the phenotype [3]. Most cases are non-syndromic, but syndromic forms—such as those associated with G/BBB syndrome, Ellis-van Creveld syndrome, Marfan syndrome, and Down syndrome—further indicate a genetic basis for this numerical anomaly [4]. Notably, Mallineni et al. [4] reported that 50% of syndromic hypohyperdontia cases were related to G/BBB syndrome.3.

The prevalence of hypohyperdontia in general population is low, ranging from 0.002% to 3.1% for non-syndromic cases [5], and approximately 0.33% in orthodontic patients [6]. This condition can affect either or both the primary and permanent dentition and can involve the maxilla, the mandible, or both jaws. Several classification systems have been proposed. Gibson [2] categorized it into premaxillary, maxillary, mandibular, and bimaxillary types. Mallineni et al. [4] simplified this into maxillary, mandibular and bimaxillary types based on occurrence. Alternatively, it can be categorized as anterior, posterior, or anteroposterior types [4].

ST exhibits a male predilection [7], can occur throughout the dental arch, and is classified based on morphology (conical, tuberculate, supplemental, and odontomes), location (mesiodens, paramolar, distomolar, and parapremolar), position (buccal, palatal, and transverse), orientation (vertical, inverted, and transverse) [7]. Orientation is defined as: vertical (long axis < 60° from vertical, root apex superior); inverted (long axis from vertical < 60°, crown superior); or transverse (long axis > 60°) [8]. Conversely, tooth agenesis affects 2.6 ~ 11.3% of the population [9], with higher prevalence in females [10]. The pattern of tooth agenesis also varies among ethnic groups. In Caucasian populations, agenesis most commonly involves the mandibular second premolar and the maxillary lateral incisor [9, 11]. In contrast, Asian populations show a higher prevalence of agenesis in the mandibular lateral incisor [9, 12, 13].

Hypohyperdontia significantly impacts oral function, aesthetics, and occlusal development, leading to reduced arch length, tooth displacement, midline deviation, and eruption disturbances, thereby complicating orthodontic and restorative management. However, due to its rarity, epidemiological data [3, 4, 14–16] remain limited in the past decade, with most reports being case studies [17–24]. Small sample sizes further constrain accurate prevalence estimation and comprehensive characterization of clinical features.

To better delineate the epidemiological and clinical characteristics of hypohyperdontia in the Chinese population, this retrospective study analyzed 10,112 panoramic radiographs (PRs) from outpatients aged 4–18 years, aiming to provide reliable prevalence data and distribution patterns to support clinical diagnosis and treatment planning.

## Methods

### Study population

This retrospective study was approved by the ethics committee of Beijing Children’s Hospital, Capital Medical University ([2025]-E-029-M). PRs were retrospectively collected from ethnic Chinese outpatients aged 4 ~ 18 years who visited the Department of Stomatology at Beijing Children’s Hospital between August 2021 and December 2023. (This study used a sample from previous research to facilitates comparative analysis of diverse dental developmental anomalies.) All the radiographic examinations were performed using a Cranex D panoramic system (Soredex, Tuusula, Finland) with standardized parameters (70 kV, 14 mA, 12-second exposure time).

### Inclusion and exclusion criteria

The inclusion criteria: (1) Pediatric dental outpatients within the specified age range; (2) Clear and undistorted PRs and first-time dental examinees.

The exclusion criteria: (1) History of permanent tooth extraction for orthodontic reasons, trauma, jaw cysts, dental caries, periodontal disease, cleft lip/palate; (2) Syndromic conditions affecting dentition; (3) distorted or blurred PRs; (4) Third molars were not counted as an event of agenesis.

### Diagnostic protocol

Prior to the formal study, diagnostic criteria were calibrated through a pilot assessment of 20 randomly selected PRs (not included in the main sample), achieving excellent inter-observer agreement (κ = 0.80). Subsequently, two calibrated clinicians independently evaluated all PRs and corresponding dental history records for concurrent ST and permanent tooth agenesis. Discrepancies were adjudicated by a third senior clinician, with final diagnosis requiring agreement from at least two examiners.

### Data collection and statistical analysis

For patients diagnosed with hypohyperdontia, the following data were collected: (1) age, gender, dentition stage; (2) characteristics of ST (number, morphology, location and orientation); (3) characteristics of tooth agenesis (number and location); (4) eruption status (pre- or post-eruption groups based on the status of anterior permanent and ST). Cases of hypohyperdontia were classified according to Mallineni et al. [4] into maxillary, mandibular, or bimaxillary types based on jaw involvement; and anterior, posterior, or anteroposterior types based on tooth location.

Qualitative data were expressed as counts (n) and percentages (%) and were statistically analysed using logistic regression and Chi-square test in SPSS (SPSS Inc. Chicago, IL) (*p* < 0.05).

## Results

Among 10,112 patients (56.00% male, 44.00% female; M:F = 1.27), the prevalence of hypohyperdontia was 0.66% (67/10112; M: F = 42:25), with no significant gender difference (OR = 1.28, 95% CI: 0.78–2.11, *p* = 0.329) (Table 1). The mean patient age was 6.97 years. Most cases (85.07%,57/67) presented after eruption of permanent anterior or ST. Hypohyperdontia was observed predominantly in the mixed dentition (68.66%,46/67), followed by primary (25.37%,17/67) and permanent (5.97%,4/67) dentitions. The anteroposterior type (58.21%,39/67) predominated over anterior type (41.79%,28/67); no posterior type was observed. Bimaxillary involvement accounted for 85.07% of cases, while maxillary and mandibular types comprised 13.43% and 1.49%, respectively (Table 2).

**Table 1:** Gender distribution of patients with hypohyperdontia

Gender	Hypohyperdontia	No Hypohyperdontia	OR	95% CI	*p*
Male	42	5621	1.28	0.78–2.11	0.329
Female	25	4424			
Total	67	10,045

**Table 2:** Type distribution of patients with hypohyperdontia according to Mallineni et al. [4]

Type	Anterior	Anteroposterior	Total
Bimaxillary	25	32	57
Maxillary	3	6	9
Mandibular	0	1	1
Total	28	39	67

### Supernumerary teeth characteristics

73.13% (49/67) of patients had 1 supernumerary tooth, while 26.87% (18/67) had 2 (χ²=14.391, *p* = 0.001). No cases exceeded 3 ST (Table 3). 67 hypohyperdontia patients exhibited 85 ST (29 maxillary mesiodens, 53 in maxillary incisor region (excluding mesiodens), 3 in the mandible). ST occurred significantly more frequently in the maxilla than mandible (65 vs. 2; χ²=59.436, *p* < 0.001), primarily in the anterior region (Table 4). Morphologically, conical (65.88%, 56/85) and tuberculate (29.41%, 25/85) types were most frequent (Table 3). By orientation, vertical (44.71%, 38/85), inverted (40.00%, 34/85), and transverse (15.29%, 13/85) alignments were observed.

**Table 3:** Morphology distribution of supernumerary teeth in patients with hypohyperdontia

	Morphology	Male	Female	Total
Single	Conical	19	11	30
	Tuberculate	13	4	17
	Supplemental	0	1	1
	odontoma	0	1	1
Double	Conical	8	5	13
	Tuberculate	2	2	4
	Supplemental	0	1	1
	odontoma	0	0	0
Total		42	25	67

**Table 4:** Location distribution of supernumerary teeth in patients with hypohyperdontia (cases/number of teeth)

Location	Male	Female	Total
maxillary mesiodens	20/22	6/7	26/29
maxillary incisor region (excluding mesiodens)	22/30	17/23	39/53
Mandible	0	2/3	2/3
Total	42/52	25/33	67/85

### Tooth agenesis characteristics

**Fig. 1:**
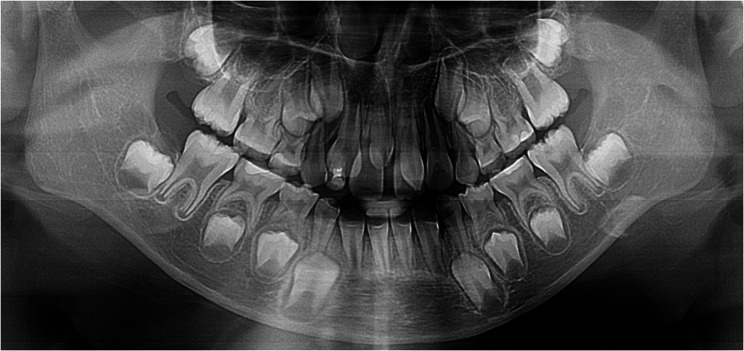
Panoramic radiograph of a 7-year-old female showing a congenitally missing bilateral maxillary second premolars, 2 supernumerary teeth located in the maxillary anterior region

**Fig. 2:**
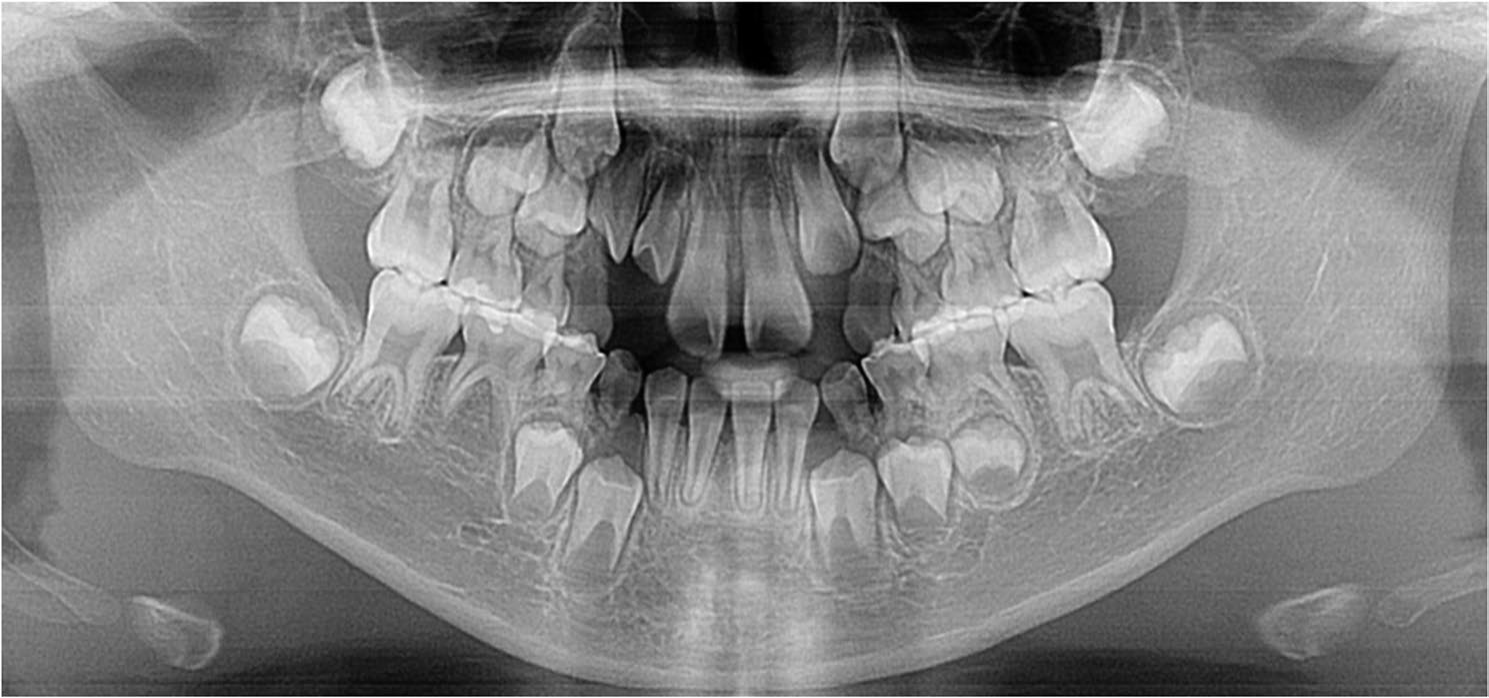
Panoramic radiograph of a 6-year-old female showing congenitally missing right mandibular second premolar, 1 supernumerary tooth located in right maxillary lateral incisor region

**Fig. 3:**
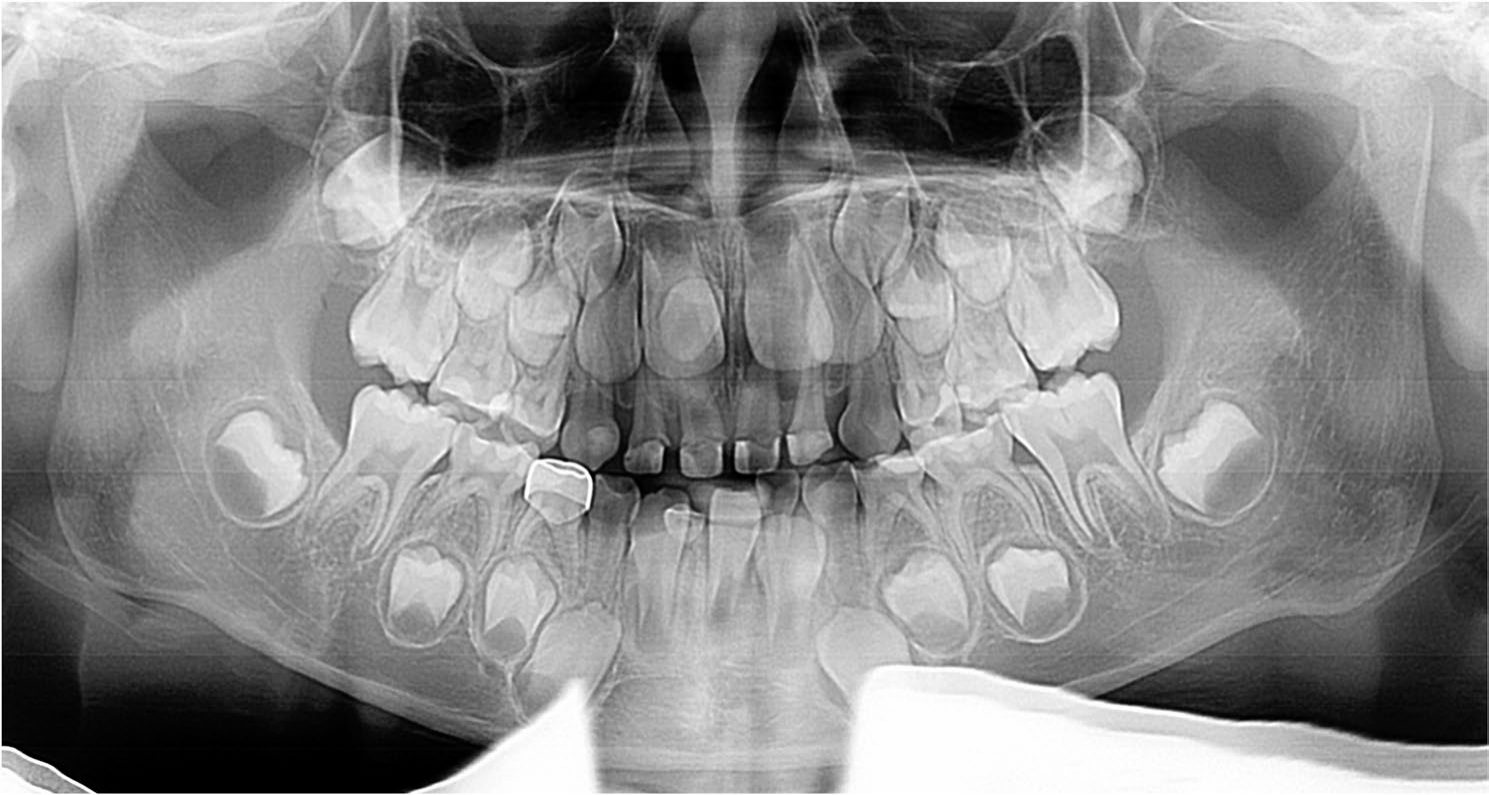
Panoramic radiograph of a 7-year-old male showing congenitally missing right mandibular lateral incisor, 1 supernumerary tooth located in maxillary anterior region

The 67 hypohyperdontia cases exhibited 115 congenitally missing teeth (mean 1.72/patient), predominantly involving 1 ~ 2 teeth (60 cases). Mandibular agenesis (81) significantly exceeded maxillary (34; χ²=19.217, *p* < 0.001), with mandibular second premolars (40) being most frequently affected, followed by maxillary second premolars (24) and mandibular lateral incisors (22). No significant laterality difference was found (left:55, right:60; χ²=0.217, *p* = 0.641) (Table 5). Figs. 1, 2, 3 are panoramic radiographs of some hypohyperdontia cases.

**Table 5:** Distribution of teeth agenesis in patients with hypohyperdontia

Teeth agenesis	Right Maxilla	Left Maxilla	Left Mandible	Right Mandible	Total
Second premolar	13	11	20	20	64
First premolar	2	1	5	1	9
Lateral incisor	2	2	9	13	26
Central incisor	0	0	6	7	13
canine	2	1	0	0	3
Total	19	15	40	41	115

## Discussion

The aetiology of hypohyperdontia remains unclear. It is generally attributed to genetic and environmental factors [3, 25–27]; however, from the current limited literature [25, 28], some authors suggest that disturbances in differentiation, migration and proliferation of neural crest cells were associated with interactions between the epithelial and the mesenchymal cells during the initiation of odontogenesis may be responsible for hypohyperdontia.

The prevalence of hypohyperdontia varies considerably (0.002%~3.1%) across studies [5], primarily due to methodological differences in sampling and diagnostic criteria. Earlier investigations that relied solely on clinical examination may have underestimated the true prevalence, while the inclusion of syndromic cases (e.g., cleft lip/palate) or third molar agenesis in some studies likely led to overestimation. In this study, third molars were not counted as an event of agenesis. Since the mean age of the first appearance of third molar bud is around 9 [29], agenesis cannot be reliably assessed. Gibson [2] emphasized that local clinical practices may further influence detection rates. Meanwhile, advancements in imaging techniques have facilitated the identification of a growing number of hypohyperdontia cases. A prevalence of 0.66% was identified in this study, with 42 cases observed in males and 25 in females. No significant gender predilection was observed, which is consistent with previous reports [5, 27]. This finding, however, contrasts with several studies that indicated a male predominance in hypohyperdontia [4, 14].

This study demonstrates that hypohyperdontia primarily manifested as the bimaxillary type (41.79% anterior, 58.21% anteroposterior), with a notable absence of posterior presentations-a finding consistent with existing literature [4, 5, 14]. The majority of cases exhibited 1 ~ 2 ST, with single conical teeth being most prevalent. The premaxillary region emerged as the most common location, while maxillary mesiodens showed significantly higher occurrence than mandibular mesiodens [4] - a pattern identical to that observed in isolated supernumerary cases [30].This study demonstrates that hypohyperdontia typically involves agenesis of 1 ~ 2 teeth, showing a significant mandibular predilection over maxillary involvement. Mandibular second premolars were most frequently absent, followed by maxillary second premolars and lateral incisors, aligning with findings by Wang et al. [3] and MSK et al. [4]. However, some authors [5, 14] have identified mandibular lateral incisors or central incisors as the most commonly affected, these variations likely reflect ethnic differences in agenesis patterns. Importantly, the observed agenesis distribution in hypohyperdontia closely paralleled that seen in isolated tooth agenesis cases [31–34].

Hypohyperdontia is typically diagnosed during mixed dentition. This study reported a mean diagnosis age of 6.97 years, consistent with Wang et al.‘s finding of 6.9 years [3]. Higher mean ages in other studies [15, 28, 35] may be associated with different sampling methods, while greater aesthetic demands likely prompted earlier visits in this study. The younger age suggests earlier detection, not earlier onset, since hypohyperdontia is often asymptomatic and discovered incidentally during other complaints. Early diagnosis and intervention are key strengths of this study.

85.07% of hypohyperdontia patients were made diagnosis following the eruption of the supernumerary or permanent anterior teeth. A delayed diagnosis is associated with an increased risk of complications, including crowding, delayed or ectopic eruption, impaction and abnormal root formation of permanent incisors, spacing, root resorption, cystic lesions, teeth rotation, and retained primary teeth [7]. Early radiographic diagnosis is therefore critical for timely intervention (or non-intervention) to prevent complications and simplify subsequent management [36]. Prior to panoramic radiography for hypohyperdontia diagnosis, early indicators—including abnormal permanent anterior tooth eruption, premature loss of primary tooth and interdental spacing —should be evaluated. For all suspected cases, panoramic radiography during the early mixed dentition stage is recommended to improve detection rates and facilitate timely intervention.

Given the lack of standardized treatment protocols, a multidisciplinary approach remains crucial for optimal management. Options include mesiodens extraction with orthodontic space closure, no treatment, immediate temporary restoration using Maryland bridges post-extraction, composite resin restoration for converting the mesiodens to mimic an incisor and implants [15, 37]. Extraction is generally indicated for most erupted ST. The optimal timing for removal of unerupted maxillary ST, however, remains controversial. Current evidence suggests that intervention during root formation stage C of the adjacent permanent teeth minimizes complications [38]. Optimal timing for extraction remains debated, with recommendations ranging from 6 to 7 years [39] to 8–10 years of age [38]. Asymptomatic cases may be retained to compensate for adjacent hypodontia. For permanent successors, most of the impacted permanent anterior teeth will erupt spontaneously post-extraction, while severely malpositioned cases (particularly those with high angulation) require orthodontic traction [40]. Combined surgical-orthodontic management is recommended due to limited spontaneous eruption potential.

This large-sample study provides a prevalence estimate and characterizes the distribution of hypohyperdontia. Identification of susceptible sites offers clinical guidance, and eruption status stratification informs diagnostic timing. However, as a retrospective outpatient study, prevalence may be inflated by selection bias. The lack of an internal control group prevents definitive conclusions regarding the uniqueness of the observed patterns. Future studies will analyze malocclusion features and treatment approaches, and explore etiology and early diagnostics markers through prospective controlled designs.

## Conclusions

Prevalence of hypohyperdontia was 0.66%, occurring mainly in the mixed dentition with bimaxillary involvement and no gender predilection. Posterior presentations were rare. Combined panoramic and clinical examinations, focusing on the anterior maxilla and second premolars/lateral incisors, is recommended for early diagnosis and complication prevention of suspected dental anomalies.

## Data Availability

All data generated or analysed during this study are included in this published article.

## Abbreviations

PR: panoramic radiograph
ST: supernumerary teeth
